# Antibiotic Residues and Antibiotic-Resistant Bacteria in Pig Slurry Used to Fertilize Agricultural Fields

**DOI:** 10.3390/antibiotics9010034

**Published:** 2020-01-17

**Authors:** Geertrui Rasschaert, Daan Van Elst, Lander Colson, Lieve Herman, Helena Cardoso de Carvalho Ferreira, Jeroen Dewulf, Johan Decrop, Jurgen Meirlaen, Marc Heyndrickx, Els Daeseleire

**Affiliations:** 1 Flanders Research Institute for Agriculture, Fisheries and Food (ILVO), 9090 Melle, Belgium; daan_van_elst@hotmail.com (D.V.E.); Lander.colson@ilvo.vlaanderen.be (L.C.); lieve.herman@ilvo.vlaanderen.be (L.H.); marc.heyndrickx@ilvo.vlaanderen.be (M.H.); els.daeseleire@ilvo.vlaanderen.be (E.D.); 2 Department of Reproduction, Obstetrics and Herd health, Faculty of Veterinary Medicine, Ghent University, 9820 Merelbeke, Belgium; helena.ferreira@ugent.be (H.C.d.C.F.); jeroen.dewulf@ugent.be (J.D.); 3 Flemish Land Agency (VLM), Manure Bank, Enforcement Nitrate Directive, 1210 Brussel, Belgium; johan.decrop@vlm.be; 4 Flemish Environmental Agency (VMM), Department of Water Reporting, 9300 Aalst, Belgium; j.meirlaen@vmm.be; 5 Department of Pathology, Bacteriology and Avian Diseases, Faculty of Veterinary Medicine, Ghent University, 9820 Merelbeke, Belgium

**Keywords:** antibiotic residues, resistance, *Salmonella*, *E. coli*, pig manure

## Abstract

Pig manure may contain antibiotic residues, antibiotic-resistant bacteria or pathogens, which may reach the environment upon fertilization. During this study, 69 antibiotic residues belonging to 12 classes were quantified in 89 pig slurry samples. These samples were also studied for the presence of *Salmonella* and for *E. coli* resistant to meropenem, colistin, ciprofloxacin, or cefotaxim. The obtained isolates were further tested for antibacterial susceptibility. No antibiotic residues were detected in four samples, whereas in the other samples, up to 12 antibiotics were found. The most frequently detected antibiotic residues were doxycycline, sulfadiazine, and lincomycin. Doxycycline was found in the highest concentration with a mean of 1476 µg/kg manure (range: 18–13632 µg/kg). Tylosin and oxytetracycline were found with mean concentrations of 784 µg/kg (range: 17–5599 µg/kg) and 482 µg/kg (range: 11–3865 µg/kg), respectively. Lincomycin, had a mean concentration of 177 µg/kg manure (range: 9–3154 µg/kg). All other 18 antibiotic residues were found with mean concentrations of less than 100 µg/kg manure. Fifty-one slurry samples harbored *Salmonella*; 35% of the *Salmonella* isolates were sensitive to a panel of 14 antibiotics, whereas the other 65% were resistant up to five antibiotics. For *E. coli*, 52 manure samples contained *E. coli* isolates which were resistant to ciprofloxacin and 22 resistant to cefotaxime. All ciprofloxacin and cefotaxime-resistant isolates were multi-resistant, with resistance up to nine and eight antibiotics, respectively. This research indicates that pig slurry used for fertilization often contains antibiotic residues and antibiotic-resistant bacteria, including pathogens.

## 1. Introduction

Belgium is known for its intensive pig farming. Almost 95% of the Belgian pigs are reared in Flanders, the northern region of Belgium, resulting in nearly 6 million pigs in a small area, with a pig density of more than 400 pigs/km^2^ [1]. The manure produced by these pigs (39.5 million kg N) is used as untreated manure to fertilize the fields (49%), exported to neighboring countries (4%), or treated in manure treatment systems followed either by use on Flemish fields or export (47%) [2].

Antibiotic use in livestock agriculture is high in many countries, including Belgium. In recent years, efforts to reduce antibiotic use in the Belgian production animals has led to a reduction in antibiotic use of 35% in 2018 as compared to 2011 [3]. Belgian studies that surveyed farmers on their use of veterinary antibiotics reveal that the most frequently used antibiotics in Belgian pig industry are amoxicillin, ceftiofur, colistin, doxycycline, and trimethoprim combined with sulfonamides [4,5].

When pigs receive antibiotics, a large fraction of these compounds is excreted unmetabolized in urine and feces [6]. Metabolism of the compounds is dependent on the oral bioavailability and the chemical structure of the antibiotic. For example, the oral bioavailability of doxycycline in pigs is reported to be between 21–50%, which is quite low and results in a high excretion of doxycycline in feces [7,8]. In most cases, manure is not applied immediately on the fields but is stored for a few weeks during summer, when the manure pit is emptied frequently, and up to 6 months during winter when it is forbidden to apply manure on the fields. In Flanders, with some exceptions, farmers are not allowed to use raw manure on the fields from 1 September to 15 February. The antibiotic concentrations ending up in the environment are therefore also dependent on the storage time in the manure pit. For example, penicillins, including the frequently used amoxicillin, degrade within a few days in manure [9]. In most of these cases, only traces of the unmetabolized product will reach the environment. After fertilization of the fields, antibiotic residues may also spread to other ecological niches. Antibiotic residues may leach to groundwater, which is also dependent on the soil type and antibiotic properties. Due to runoff, residues may enter surface water and watercourses. Antibiotic residues may be taken up by crops growing in soils fertilized with manure or irrigated with water containing antibiotic residues [10].

Antibiotic use in human and veterinary medicine is considered as the key driver for the current antibiotic resistance selection. Correlation between antibiotic use and resistance has been demonstrated both in the human and the veterinary sector [11,12]. Awareness is growing that very low antibiotic concentrations, up to several hundredfold below the Minimum Inhibitory Concentration (MIC), are also able to select for antibiotic resistance [13,14]. Upon antibiotic use in animals, antibiotic-resistant bacteria may be selected in the gastrointestinal tract of the animals itself as well as in different environmental niches such as fertilized fields, groundwater, surface water and watercourses and possibly even on crops. Furthermore, in each ecological niche, these antibiotic-resistant bacteria may—when the antibiotic resistance gene is located on a mobile element—pass these resistant genes to susceptible bacteria.

Pig manure may harbor not only antibiotic residues and antibiotic-resistant bacteria such as the indicator organism *E. coli*, but also pathogens. For human health, zoonoses are especially important to consider. Since 2005 *Salmonella* has become the second most important reported zoonosis in the EU. Besides poultry, pigs are also considered as important reservoir of *Salmonella*, especially for the serovar *Salmonella* Typhimurium [15]. Pigs infected with *Salmonella* are usually not symptomatic but may excrete the bacteria intermittently via their feces. Further, *Salmonella* may survive several months in pig manure [16]. Last, *Salmonella* and especially *Salmonella* Typhimurium, may be quite resistant to antibiotics.

All ecological niches are connected according to the One Health Concept. Bacteria, including pathogens and antibiotic-resistant bacteria, and antibiotic residues may reach livestock or humans when raw manure is spread on arable lands. Eventually, this may lead to diseases difficult to treat for both animals and humans.

According to the risk-assessment of de la Torre et al. [17], Belgium is one of the top three European countries with the highest risk for antibiotic soil contamination. The present study aimed to determine the concentrations of antibiotics in pig manure used to fertilize agricultural fields. Furthermore, the prevalence of *Salmonella* in manure samples was determined as well as the antibiotic resistance of the obtained isolates. Last, the antibiotic resistance of *E. coli* as indicator organism was studied. As all manure samples were expected to contain high *E. coli* levels and because we wanted to investigate if manure contained *E. coli* bacteria resistant to antibiotics considered as critically important in human medicine, manure was plated on plates containing meropenem, colistine, ciprofloxacin, or cefotaxime [18].

## 2. Results

### 2.1. Antibiotic Residues

**Figure 1:**
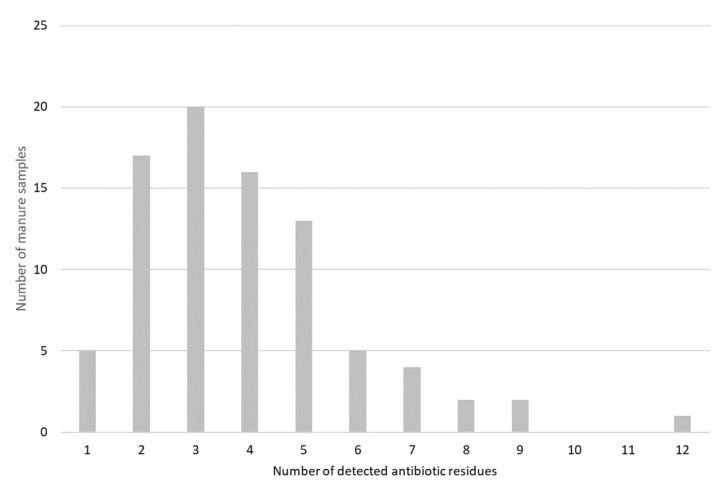
The number of pig manure samples relative to the number of detected antibiotic residues (*n* = 85).

In 85 out of the 89 manure samples antibiotic residues were detected. In total, 23 different antibiotic residues were detected. In the majority of the samples (76 samples), between 1 and 6 different antibiotic residues were found. In one sample, 12 different antibiotics were detected (Figure 1).

The most frequently detected antibiotic residues in the manure samples were doxycycline (82.0%), sulfadiazine (70.8%) and lincomycin (69.7%) (Table 1). Besides being the most frequently detected antibiotic, doxycycline was also found in the highest concentration, with a mean of 1475.8 µg/kg manure. In 6 manure samples, doxycycline concentrations higher than 5000 µg/kg manure were detected of which one higher than 10000 µg/kg manure. Tylosin, which was detected in 11.2% of the samples, was found in the second highest concentration (784.3 µg/kg manure). Oxytetracycline, with the third highest average concentration, was found in a mean concentration of 481.9 µg/kg manure. Lincomycin, the third most frequently detected antibiotic with detection in 69.7% of the pig manure samples, had a mean concentration of 176.7 µg/kg manure. Colistin was only detected in one sample in a concentration of 116 µg/kg. All other 18 antibiotic residues were found with mean concentrations of less than 100 µg/kg manure (Table 1). Sulfadiazine, which was the second most detected antibiotic residue, had a mean concentration of 60.7 µg/kg manure. In 36 out of the 63 manure samples containing sulfadiazine, the concentration was below LOQ.

### 2.2. Salmonella, E. coli and Antibiotic Resistance

Of the 89 manure samples, 51 samples (57.3%) harbored *Salmonella*. Of these *Salmonella* isolates (*n* = 51), 22 were identified as *Salmonella* Typhimurium (43.1%). Antimicrobial resistance profiling revealed that 11.8% were resistant to one antibiotic, 11.8% to two antibiotics, 17.6% to three antibiotics, 13.6% to four antibiotics and 9.8% to five antibiotics. The remaining 18 isolates of these 51 *Salmonella* isolates (35.2%) were sensitive for all 14 tested antibiotics. High antibiotic resistance rates were observed for ampicillin (54.7%), sulfamethoxazole (47.2%) and tetracycline (45.3%). In addition, resistance to ampicillin, sulfamethoxazole and tetracycline was the most frequent resistance profile; this was either observed as resistance to only those three antibiotics or in combination with resistance to chloramphenicol, trimethoprim and/or gentamicin (Table 2 and Table 3).

For *E. coli*, 52 of the 89 manure samples (58.4%) contained *E. coli* isolates resistant to ciprofloxacin in a mean concentration of 3.0 log cfu/g manure. Cefotaxim-resistant *E. coli* isolates were detected in 22 samples (24.7%) in a mean count of 2.2 cfu/g manure (Table 4). None of the manure samples seemed initially to contain *E. coli* isolates resistant to colistin or meropenem. Regarding the ciprofloxacin-resistant *E. coli* isolates, the isolates also showed a high resistance to nalidixic acid (92.3%), sulfamethoxazole (90.4%), ampicillin (78.8%), trimethoprim (73.1%) and tetracycline (59.6%). Sensititre confirmed the resistance of the cefotaxime-resistant *E. coli* isolates, but also identified one isolate resistant to colistin (with a MIC of 8 µg/mL) although the agar plates containing colistin were all negative, as mentioned above. A high resistance among these cefotaxime-resistant *E. coli* isolates was observed for the two other β-lactam antibiotics, namely ampicillin (100%) and ceftazidime (95.5%). Further, as for the ciprofloxacin-resistant *E. coli* isolates, a high resistance was found for trimethoprim (72.7%) and tetracycline (59.1%). All isolates were multi-resistant; for the ciprofloxacin-resistant strains, resistance up to nine different antibiotics was noticed. The most observed antibiotic resistance profile was resistance to ampicillin, chloramphenicol, ciprofloxacin, nalidixic acid, sulfamethoxazole, tetracycline, and trimethoprim, either alone or in combination with resistance to cefotaxim, gentamicin and/or azithromycin (Table 5). Concerning the cefotaxime-resistant *E. coli* isolates (Table 6), the most detected antibiotic resistance phenotype was resistance to ampicillin, cefotaxim, ceftazidime, tetracycline and trimethoprim.

Statistical analyses revealed no association between the presence of antibiotic residues and the presence of ciprofloxacin or cefotaxime-resistant *E. coli* isolates. For *Salmonella*, no association was found between the presence of antibiotic residues and resistance to the antibiotics tested in the Sensititre EU Surveillance *Salmonella*/*E. coli* (EUVSEC) plate (Thermo Fisher Scientific, Waltham, MA, USA) with the exception of an association between the presence of fluoroquinolones and resistance to trimethoprim (*p* = 0.02).

## 3. Discussion

This study clearly demonstrates that raw manure often contains antibiotic residues when it is spread on the fields. The samples were taken from February to May, in the period that farmers fertilize the soils after the winter and when most manure pits are nearly completely filled. This means that most slurry samples contained a mixture of manure of different ages ranging between 6 months old and fresh manure. This also implies that this study is not suitable for drawing conclusions about the stability of the residues in the manure pit and survival of pathogens and antibiotic-resistant bacteria.

Despite many efforts to reduce the veterinary antibiotic use, it is remarkable that nearly all manure samples contained antibiotic residues. As reported already previously [9,19], we were also not able to detect any of the penicillins due to fast degradation in manure caused by the hydrolysis of the β-lactam ring. Pig farmers that shared data about the antibiotic use in the months before the sampling reported the use of the penicillins amoxicillin, ampicillin, and benzylpenicillin. However, as we were also not able to detect any of the other listed β-lactam antibiotics, we are not certain if this is due to degradation of these antibiotics or that these antibiotics were not used. For example, ceftiofur is known to be used in the pig industry but none of the surveyed farmers mentioned having used it. The fast degradation of these antibiotics does not mean that they have no impact on the resistance, as they can exert a pressure on the microbiota in the animal itself during treatment. Further, resistance to β-lactam antibiotics can be co-selected by the use of other antibiotics [20].

With an occurrence of 82%, doxycycline is the antibiotic that was most frequently detected in raw manure. In addition, it was also detected in the highest concentration, with a mean of 1.5 mg/kg manure and a maximum concentration of more than 10 mg/kg manure. This high occurrence and the observed high concentrations are in agreement with other reports from Belgium and The Netherlands [9,19]. Oxytetracycline was found in the third highest mean concentration (approximately 0.5 mg/kg manure) but was detected less frequently compared to doxycycline. The other two tetracyclines were of lesser importance, both in terms of occurrence as in concentration. It can be roughly estimated that the manure and the antibiotic residues are diluted by a factor 100–150 when mixed with the top layers of the soil ([21], personal communication with Johan De Crop of the Flemish Manure Bank). Manure is not evenly homogenized in the soil, however, which results in some areas with more concentrated manure. The main question is if the diluted antibiotic concentrations, which for doxycycline are expected to range from 0.12 to 140 µg/kg soil with a mean of 10–15 µg/kg, can exert an impact on the antibiotic resistance in the environment. Based on several experimental or modeling studies, we may assume that for tetracycline and especially doxycycline, this might be the case. First, the sorption of the antibiotic to the soil and the half-life of the antibiotic in the soil, which are both dependent on the antibiotic and the soil type are important but difficult to predict factors. However, it has been described that tetracyclines are strongly adsorbed to the soil and are persistent in both manure and soil and do not degrade quickly [22,23]. Second, Gullberg et al. [13] demonstrated for tetracycline in in vitro experiments that 15 µg/L (i.e., 1/100 of the MIC) may select for resistant bacteria. This value corresponds approximately with our expected mean concentration of doxycycline in the soil. Further, according to Bengtsson-Palme et al. [24] who modeled the Predicted No Effect Concentrations (PNECs) for a range of antibiotics as indicator for resistance selection, doxycycline concentrations above 2 µg/L may promote antibiotic resistance. This implies for the present study that 75% of the fertilized soils contained doxycycline concentrations might be able to exert antibiotic resistance in the soil. Surely, in vitro studies or modeling studies are not an exact representation of the complex soil microbiome. In addition, the bioavailability of antibiotics in soil may be lower in soil than in other matrices. So, field experiments are urgently needed to study the impact of such low doses. Yan et al. [25] showed that the soil microbiome and certain tetracycline-resistant genes were altered due to fertilization with manure containing doxycycline in the range of 4.4 to 13.2 mg per kg soil. However, in that study the manure was spiked with much higher doxycycline concentrations than found in the present manure samples.

Sulfadiazine was the second most frequently detected antibiotic, although the observed concentrations were quite low; 75% of the samples had sulfadiazine concentrations below 10 µg/kg and in more than half of the samples concentrations below the LOD were found. It was demonstrated before that sulfonamides dissipate quickly in manure [22] which is in agreement with our results. Sulfonamides are easily detected and quantified in manure by means of LC-MS/MS, however, which explains the frequent detection of sulfadiazine in the samples and the low LOD and LOQ in the present study. Considering the dilution factor of 100–150 in soil after fertilization, one can assume that the sulfadiazine concentrations in the soil are generally very low. Despite the fast degradation in manure, several studies demonstrated that of all studied antibiotic classes, the sulfonamides are often the most frequently detected antibiotics in groundwater in livestock areas which can be explained by leaching of these antibiotics from the topsoil to the lower levels due to their high water solubility [10,26].

The high detection rate of lincomycin in the manure samples is probably due to the high persistence of lincomycin in manure: it can be detected in pig manure for more than one year [22]. It can be assumed that this antibiotic can often been found in the soil after fertilization but in rather low concentration, as 75% of the manure samples contained concentrations below 80 µg/kg manure and 50% of the samples contained concentrations below approximately 20 µg/kg manure. Using the 100–150-fold dilution factor mentioned above, it can be estimated that with some exceptions, concentrations between 0.1–1 µg/kg manure can be found in the soil upon fertilization. The PNEC has been calculated to be 2 µg/L so in this case, in only a minority of the samples, this theoretical threshold is exceeded. As for the sulfonamides, the water-solubility is quite high and therefore lincomycin can leach to the groundwater level as demonstrated before [10]. Again, besides a theoretical PNEC value, it is not clear yet what the impact of these antibiotics are on antimicrobial resistance.

It was quite remarkable that more than half of the samples still contained *Salmonella*, which indicates that *Salmonella* is still a problem in pig farming. This prevalence is considerably higher than the levels reported before [27]. Although some studies report that *Salmonella* can survive up to four months on the soils upon fertilization, it can be assumed that the majority of the manure-associated bacteria including pathogens die off within two weeks after fertilization [28]. It cannot be excluded that this pathogen is further spread by wild fauna, e.g., by wild birds picking grains after sowing. It is also the possible risk of the further spread of resistance genes to soil-associated bacteria as 65% of the *Salmonella* isolates were resistant to antibiotics and approx. 40% of isolates were resistant to three or more antibiotics.

As the risk exists that resistance genes reach people directly via exposure with the environment, or indirectly when resistance genes are passed to bacteria in the environment or crops, we wanted to know if manure contained bacteria, such as *E. coli*, that are resistant to antibiotics used in human medicine, especially those as considered as critically important [18]. We chose meropenem, ciprofloxacin, cefotaxime as representative antibiotics of different antibiotic classes and because they all very important in human health. Colistin is also considered as last-resort antibiotic in human health, due to its nephrotoxic effects. However, this antibiotic is still used in Belgian pig farming to treat post-weaning diarrhea despite the introduction of zinc oxide as an alternative [3]. Surprisingly, no colistin-resistant *E. coli* bacteria were found when plated out on colistin-spiked plates, although one of the cefotaxime-resistant *E. coli* isolates was resistant to colistin when tested with Sensititre. Also, none of the *Salmonella* isolates was resistant to colistin. This observed resistance rates are overall in agreement with the European Food Safety Authority (EFSA) data [29] for *Salmonella* isolated from pig carcasses in Belgium. For *E. coli*, a comparison with other studies is difficult as in our study, the *E. coli* isolates were preselected on antibiotic containing plates, unlike the testing performed in monitoring programs. For example, in our study 58% of the slurry samples contained ciprofloxacin-resistant *E. coli* bacteria and 25% of the samples contained cefotaxime-resistant *E. coli* bacteria. In the EFSA study [29], for both antibiotics this resistance was low (1–10%) in Belgian pigs.

No associations were found between the presence of antibiotic residues, grouped in antibiotic classes, and the presence of cefotaxime or ciprofloxacin-resistant *E. coli* isolates. An association between the presence of cephalosporins and the occurrence of cefotaxime-resistant *E. coli* could not be determined as cephalosporines were not be detected, probably due to chemical breakdown of β-lactam antibiotics as described above. However, an association between the presence of fluoroquinolones and the presence of ciprofloxacin-resistant *E. coli* isolates was also lacking. When an antibiotic class is not detected, this does not mean that it is or was not present. It can be present below the detection limit at the time of analysis or it can be degraded. An association was, however, detected between the presence of fluoroquinolones and resistance to trimethoprim in *Salmonella*. As the underlying mechanism for this resistance was not determined, it is difficult to determine whether this is truly reflecting a biologically meaningful association or rather a type 2 error linked to the multiple associations tested.

In summary, this study clearly demonstrates that raw pig slurry contains antibiotic residues and antibiotic-resistant bacteria, including pathogens as *Salmonella*, at the time manure is used as fertilizer. Further research should study the fate of these antibiotic concentrations, especially doxycycline, sulfadiazine and lincomycin, and their possible effect on the induction or spread of antibiotic resistance in the environment. In addition, more research should be focused on the effect of manure treatment on the antibiotic residues and resistant bacteria.

## 4. Materials and Methods

### 4.1. Sample Collection

In total, 89 samples of pig manure were collected in Flanders (the northern part of Belgium, which is characterized by intensive pig farming). Samples consisted of manure from fattening pigs or from sows and piglets. The samples were collected between the 16 February 2017, the legal start date that farmers are allowed to deposit manure on agricultural fields, and the 4 May 2017. They were collected by the Flemish Land Agency, which monitors the correct implementation of the environmental manure legislation. Annually they take approximately 800 manure samples for nitrogen and phosphorus analysis. Sample collection was performed when (i) the manure pit was emptied for deposition on the fields, or (ii) after transport, just before fields were fertilized with the manure, or (iii) at arrival at the manure treatment plant. Some farmers chose for the latter option in February and March 2017 when the manure pits were completely filled, and they were not able to fertilize the fields due to the rainy weather in that period. The homogenization of the manure was guaranteed as the samples were collected according to BAM/part3/01 (described in Flemish law of 16 July 2010 regarding the protection of the environment by pollution of fertilizers). All samples were stored at 4 °C and transported to the laboratory within four days after sampling. At arrival at the laboratory, the samples were homogenized and portions of 5 g and 2 g were frozen (−21 °C) in polypropylene (PP) tubes for later chemical analysis. Bacterial analysis was started immediately upon arrival.

### 4.2. Antibiotic Residues

In total, the manure samples were screened for the presence of 69 antibiotic residues (Table 7). This was performed in three different extraction protocols and runs. The first method, called the multi-residue method, is suitable to detect and quantify antibiotics belonging to the following classes: β-lactam antibiotics, tetracyclines, macrolides, (fluoro) quinolones, sulfonamides and trimethoprim, pleuromutilins, amphenicols, lincosamides and diaminopyrimidine derivates. The second method is appropriate for the aminoglycosides and the third one for colistin.

For the multi-residue method, the protocol for the extraction and mass-spectrometry was performed as described by Van den Meersche et al. [19] (2016) with some minor modifications. First, the residue was redissolved in 1 mL of water/acetonitrile (80/20, *v*/*v*). Second, a higher number of antibiotics were analyzed than described by Van den Meersche et al. [19] but colistin was analyzed in a separate analysis which is described below (Table 8). Consequently, the samples were spiked with several other internal standards, namely cincophen, trimethoprim-d9, sulfadimethoxine ^13^C_6_, lomefloxacine, clindamycin, methacycline, cefotaxime, ceftiofur-d3, piperacillin, roxithromycin and chloramphenicol-d5. As a lot of antibiotics were analyzed in this method, a screening method was applied, meaning that the transition of the precursor ion to only one product ion was followed (Table 1). In case a signal was obtained, the sample was reinjected and minimum 2 product ions were followed. The Limit of Detection (LOD) was calculated according to the following formula: 3 × (SD/S), with SD being the standard deviation of the response based on the standard deviation of y-intercepts of regression lines and S being the slope of the calibration curve. The Limit of Quantification (LOQ) was calculated in an analogous way but with the formula 10 × (SD/S). The expanded measurement uncertainty was calculated using data of 4 samples spiked at 5, 25 and 50 µg/kg, respectively. The limit of the detection (LOD), the limit of quantification (LOQ) and the expanded measurement uncertainty are shown in Table 2. For most β-lactam antibiotics no data could be obtained from the validation study. Probably the concentration levels of the calibration curve (0 to 100 µg/kg) were too low for those compounds. When analyzing the samples of the study described, the antibiotics were spiked at 500 µg/kg; for many samples at this level, a good signal in the chromatograms could be obtained. Manure composition may also play an important role, in particular the presence or absence of enzymes that can break down the penicillins.

For the extraction of the aminoglycosides, a 5 g subsample was thawed and the internal standard ribostamycin was added. The spiked sample was left to equilibrate at room temperature for 10 min. After addition of 15 mL of trichloroacetic acid (20%) the tube was placed on a shaker during 30 min. Afterward the sample was centrifuged for 15 min at 4700× *g*. The supernatant was further purified over OASIS HLB solid phase extraction columns. The extract was filtered through a 0.22 µm filter and 10 µL was injected in the LC-MS/MS system (Acquity UHPLC, column: Obelisc R (Sielc) (2.1 × 100 mm; 5 µm, 100 Å) and analogous pre-column, solvent 1: water + 0.1% formic acid, solvent 2: acetonitrile + 0.1% formic acid). The LOD and LOQ were determined as described above. The linearity was assessed using two calibration curves with six calibration points in a concentration range of 0 to 2000 µg/kg (to 4000 µg/kg for streptomycin and to 8000 µg/kg for spectinomycin). For the recovery, repeatability and reproducibility, blank manure was spiked with pure analytical standards in three different concentrations (Table 9). The expanded measurement uncertainty was calculated with coverage factor 2. The obtained results for this validation are shown in Table 3.

When an antibiotic residue was detected below the LOQ, then the value of the LOQ was used for the further interpretation.

For the extraction of the colistin, a 5 g subsample was thawed and the internal standard polymyxin B was added. The spiked sample was left to equilibrate at room temperature for 10 min. After addition of 5 mL of extraction solvent (20% trichloroacetic acid) and 5 mL of water, the sample was vortex mixed and put on a shaker during 10 min at 250 rpm. After centrifugation at 4000× *g* during 15 min, 10 mL of the supernatant was collected in a TTP tube. Ten milliliters of 20 mM K2HPO4 solution and 2 × 150 µL NaOH (30%) was added, with control of pH (7) between steps. After centrifugation, the extract was brought onto a WP-CBX SPE (6 mL, 500 mg) column from Bakerbond that was conditioned with 2 × 6 mL methanol, 6 mL water and 6 mL of 20 mM K2HPO4 solution. After passing through the feces extract, the column was washed with 6 mL of water. After drying, the residues were eluted with 2 × 3 mL of a solution of 10% formic acid in methanol. The eluate was evaporated under nitrogen at 60 °C and the residues were dissolved in 1 mL acetonitrile/water (50/50, *v*/*v*) with 1% formic acid. The extract was filtered through a 0.22 µm filter and 10 µL was injected into the LC-MS/MS system (Acquity UHPLC, column: Atlantis HILIC silica (2.1 × 100 mm; 3 µm) and analogous pre-column, solvent 1: water, solvent 2: acetonitrile + 1% formic acid). This method was not validated but the LOD is estimated at 25 µg/kg.

When analyzing the manure samples, we observed that a quantification that made use of matrix matched calibration curves was not possible due to the high variation observed in the composition of the manure samples. In theory a standard addition for each sample should have been performed but this was not feasible for practical and financial reasons. We therefore decided to work with a one-point standard addition. Each sample was analyzed as is, plus with the addition of the antibiotics at 500 µg/kg. This concentration was chosen in the absence of information about the likely concentrations in real samples. Only semi-quantitative results were generated for the manure samples.

### 4.3. Salmonella, E. coli and Antibiotic Resistance

After homogenization, 25 g of the pig manure was added to 225 mL of Buffered Peptone Water (BPW, Oxoid CM0509, Basingstoke, UK). For *E. coli* isolation, the BPW was diluted 10 times in peptone water (Oxoid CM0009, Basingstoke, UK). From both the mother suspension and the tenfold dilution, 100 µL was immediately plated on RAPID’ *E. coli* 2 plates (Bio-Rad 356-4024, Marnes-la-Coquette, France) containing meropenem (Sigma–Aldrich PHR1772, Saint Louis, MO, USA), colistin (Sigma–Aldrich C4461, Saint Louis, MO, USA), ciprofloxacin (Sigma–Aldrich 17850, MO, USA) or cefotaxime (Sigma–Aldrich C7912, Saint Louis, MO, USA) according to the ‘The European Committee on Antimicrobial Susceptibility Testing (EUCAST)’ cut-off concentrations. These cut-off values are 0.125 mg/L for meropenem, 2 mg/L for colistin, 0.064 mg/L for ciprofloxacin and 0.25 mg/L for cefotaxim. In addition, 100 µL was also plated on RAPID’ *E. coli* 2 plates without antibiotics. After incubation at 44 °C for 24 h, colonies with a typical appearance for *E. coli* were counted. Isolates resistant to one of the four tested antibiotics were further purified and stored at −80 °C for further analysis (one isolate per sample).

For *Salmonella* isolation, the remaining BPW was incubated at 37 °C for 18 h, 100 μL was plated on the center of Modified Semi-solid Rappaport Vassiliadis agar plates supplemented with novobiocin (MSRV, Oxoid CM0910 + SR0161). After incubation for 24–48 h at 41.5 °C, a loopful from the edge of the white migration zones was plated onto xylose lysine deoxycholate agar plates (XLD, Oxoid CM469). Plates were evaluated for typical colonies of *Salmonella* after 24 h of incubation at 37 °C. From each plate, one *Salmonella* suspect colony was further purified and stored at −80 °C for further analysis. A multiplex PCR was performed to confirm the *Salmonella* genus and identify the *Salmonella* Typhimurium serotype. Primers, reaction mixture and amplification protocol were as described previously [30,31,32].

All *Salmonella* isolates and the stored *E. coli* isolates were further characterized for antimicrobial susceptibility by Sensititre micro broth dilution analysis with EUVSEC plates (ThermoFisher, West Sussex, UK) by means of the Sensititre141 ^®^ Vizion^®^ system (ThermoFisher™, West Sussex, UK) according to EURL-AR (EURL-AR 2013) guidelines. The minimum inhibitory concentration (MIC) for each of the following antimicrobials was determined: ampicillin, cefotaxime, ceftazidime, meropenem, nalidixic acid, ciprofloxacin, tetracycline, colistin, gentamicin, trimethoprim, sulfamethoxazole, chloramphenicol, azithromycin and tigecycline. Isolates were considered resistant or susceptible based on the cut-off values given in the EURL-AR guidelines (EURL-AR 2013).

### 4.4. Statistical Analysis

First, concentrations of antibiotic residues were converted to absence or presence of the antibiotic residue. Second, antibiotic residues were grouped together within their class: β-lactam antibiotics (penicilins), β-lactam antibiotics (cephalosporins), tetracycylines, macrolides, pleuomutilines, quinolones, fluoroquinolones, lincosamides, sulfonamides, trimethoprim, amphenicols, diaminopyrimidine derivates, aminoglycosides, and polymyxins.

For those antimicrobial classes where residues were found, the association between the presence of the antibiotic residue and the presence of ciprofloxacin-resistant *E. coli* or cefotaxime-resistant *E. coli* isolates was tested by means of a χ2 test. Further, in the *Salmonella* positive samples, the association between the presence of antibiotic residues and the resistance as determined by Sensititre in *Salmonella* was evaluated using the same test. All analyses were performed in Microsoft Excel^®^. A difference was assumed to be significant when *p* < 0.05.

## Figures and Tables

**Table 1:** Overview of the concentration of the detected antibiotic residues in the pig manure samples where antibiotic residues were detected (*n* = 85) (all in µg/kg).

Antibiotic Residue	No. of Times Detected (%)	Mean	Median	Minimum	Maximum	First Quartile	Third Quartile
doxycycline	73 (82.0%)	1475.8	672.0	17.9	13632.1	233.4	1753.0
tylosin	10 (11.2%)	784.3	163.2	17.3	5599.0	97.5	201.9
oxytetracycline	16 (18.0%)	481.9	59.0	11.3	3864.7	22.7	231.0
lincomycin	62 (69.7%)	176.7	21.9	9.0	3153.8	9.0	80.3
colistin	1 (1.1%)	116.1	116.1	116.1	116.1	116.1	116.1
paromomycin	1 (1.1%)	97.0	97.0	97.0	97.0	97.0	97.0
flumequine	16 (18.0%)	85.9	6.9	2.5	786.9	2.7	40.5
gentamicin	1 (1.1%)	72.0	72.0	72.0	72.0	72.0	72.0
sulfadiazine	63 (70.8%)	60.7	3.5	3.5	1358.8	3.5	8.5
tulathromycin	5 (5.6%)	45.2	37.5	33.3	80.3	33.3	41.5
tiamulin	13 (14.6%)	40.9	29.8	16.7	120.5	16.7	36.3
chlortetracycline	2 (2.2%)	35.9	35.9	13.3	58.5	24.6	47.2
tilmicosin	20 (22.5%)	25.3	8.3	8.0	221.3	8.0	11.6
marbofloxacin	16 (18.0%)	18.7	6.7	5.8	112.0	5.8	11.1
tetracycline	3 (3.4%)	16.1	12.6	9.9	25.7	11.3	19.2
enrofloxacin	10 (11.2%)	14.2	9.1	6.2	33.5	6.3	17.5
gamithromycin	1 (1.1%)	10.2	10.2	10.2	10.2	10.2	10.2
tylvalosin	1 (1.1%)	8.1	8.1	8.1	8.1	8.1	8.1
ciprofloxacin	7 (7.9%)	6.3	5.2	5.2	8.1	5.2	7.6
danofloxacin	1 (1.1%)	5.9	5.9	5.9	5.9	5.9	5.9
sulfadoxine	4 (4.5%)	5.4	3.8	2.7	11.2	2.7	6.4
trimethoprim	1 (1.1%)	4.3	4.3	4.3	4.3	4.3	4.3
sulfamethazine	5 (5.6%)	3.0	3.0	3.0	3.0	3.0	3.0

**Table 2:** Number of *Salmonella* isolates resistant to the antibiotics tested in the Sensititre EU Surveillance *Salmonella*/*E. coli* (EUVSEC) panel.

Antibiotic	No. of Resistant *Salmonella* Isolates (%)
Ampicillin	29 (54.7%)
Sulfamethoxazole	25 (47.2%)
Tetracycline	24 (45.3%)
Trimethoprim	12 (22.6%)
Chloramphenicol	7 (13.2%)
Gentamicin	1 (1.9%)
Azithromycin	0
Cefotaxime	0
Ceftazidime	0
Ciprofloxacin	0
Colistin	0
Meropenem	0
Nalidixic Acid	0
Tigecycline	0

**Table 3:** Antimicrobial resistance phenotypes of *Salmonella* isolated from pig manure.

Antibiotic Resistance Profile ^a^	No. of Isolates (%)
AMP&CHL&SMX&TET&TMP	4 (7.8%)
AMP&CHL&GEN&TET&TMP	1 (2.0%)
AMP&SMX&TET&TMP	5 (9.8%)
AMP&CHL&SMX&TET	2 (3.8%)
AMP&SMX&TET	9 (17.6%)
AMP&SMX	4 (7.8%)
AMP&TET	1 (2.0%)
SMX&TMP	1 (2.0%)
AMP	3 (5.9%)
TET	2 (3.9%)
TMP	1 (2.0%)
sensitive	18 (35.2%)
**Total**	**51 (100%)**

^a^ AMP = ampicillin, CHL = chloramphenicol, GEN = Gentamicin, TET = tetracycline, TMP = trimethoprim, SMX = sulfamethoxazole.

**Table 4:** Number and percentage of ciprofloxacin or cefotaxime-resistant *E. coli* isolates resistant to the antibiotics tested in the EUVSEC panel.

Antibiotic	Number of Ciprofloxacin-Resistant *E. coli* Isolates Resistant to the Antibiotic Listed (%)	Number of Cefotaxime-resistant *E. coli* Isolates Resistant to the Antibiotic Listed (%)
Ampicillin	41 (78.8%)	22 (100.0%)
Azithromycin	4 (7.7%)	4 (18.2%)
Cefotaxime	3 (5.8%)	**22 (100.0%)**
Ceftazidime	1 (1.9%)	21 (95.5%)
Chloramphenicol	22 (42.3%)	3 (13.6%)
Ciprofloxacin	**52 (100.0%)**	7 (31.8%)
Colistin	0	1 (4.5%)
Gentamicin	3 (5.8%)	0
Meropenem	0	0
Nalidixic Acid	48 (92.3%)	4 (18.2%)
Sulfamethoxazole	47 (90.4%)	4 (18.2%)
Tetracycline	31 (59.6%)	13 (59.1%)
Tigecycline	0	0
Trimethoprim	38 (73.1%)	16 (72.7%)

**Table 5:** Number and percentage of ciprofloxacin-resistant *E. coli* isolates resistant to the antibiotics tested in the EUVSEC panel.

Antibiotic Resistance Profile ^a^	No. of Isolates (%)
AMP&AZI&CHL&CIP&NAL&SMX&TET&TMP	2 (3.8)
AMP&CHL&CIP&GEN&NAL&SMX&TET&TMP	2 (3.8)
AMP&FOT&CHL&CIP&NAL&SMX&TET&TMP	1 (1.9)
AMP&CHL&CIP&NAL&SMX&TET&TMP	8 (15.4)
AMP&AZI&CIP&NAL&SMX&TET&TMP	1 (1.9)
AMP&FOT&CIP&NAL&SMX&TET&TMP	1 (1.9)
AMP&CHL&CIP&NAL&SMX&TMP	4 (7.7)
AMP&CIP&NAL&SMX&TET&TMP	4 (7.7)
AMP&CIP&GEN&NAL&SMX&TMP	1 (1.9)
AMP&CIP&NAL&SMX&TET	6 (11.5)
AMP&CIP&NAL&SMX&TMP	4 (7.7)
CHL&CIP&NAL&SMX&TMP	2 (3.8)
AMP&CHL&CIP&NAL&TMP	1 (1.9)
AZI&CIP&NAL&SMX&TET	1 (1.9)
AMP&CIP&NAL&SMX	2 (3.8)
CIP&NAL&SMX&TET	2 (3.8)
CIP&NAL&SMX&TMP	2 (3.8)
AMP&CHL&CIP&TMP	1 (1.9)
AMP&CIP&TET&TMP	1 (1.9)
CIP&NAL&SMX	3 (5.8)
AMP&CIP&TMP	1 (1.9)
CIP&TET&TMP	1 (1.9)

^a^ AMP = ampicillin, AZI = Azithromycin, CEF = Cefotaxim, CIP = ciprofloxacin, CHL = chloramphenicol, GEN = Gentamicin, NAL = Nalidixic Acid, TET=tetracycline, TAZ = Ceftazidime, TMP = trimethoprim, SMX = sulfamethoxazole.

**Table 6:** Number and percentage of cefotaxime-resistant *E. coli* isolates resistant to the antibiotics tested in the EUVSEC panel.

Antibiotic Resistance Profile ^a^	No. of Isolates (%)
AMP&FOT&TAZ&CIP&NAL&SMX&TET&TMP	2 (9.1)
AMP&AZI&FOT&TAZ&CIP&NAL&SMX&TMP	1 (4.5)
AMP&AZI&FOT&TAZ&CHL&TET&TMP	1 (4.5)
AMP&FOT&TAZ&CIP&NAL&SMX&TMP	1 (4.5)
AMP&AZI&FOT&TAZ&TET&TMP	1 (4.5)
AMP&FOT&TAZ&CIP&COL&TMP	1 (4.5)
AMP&FOT&TAZ&CIP&TET&TMP	1 (4.5)
AMP&FOT&TAZ&TET&TMP	3 (13.6)
AMP&AZI&FOT&TAZ&TET	1 (4.5)
AMP&FOT&TAZ&CHL&TET	1 (4.5)
AMP&FOT&TAZ&CHL&TMP	1 (4.5)
AMP&FOT&TAZ&CIP&TET	1 (4.5)
AMP&FOT&TAZ&TMP	3 (13.6)
AMP&FOT&TAZ&TET	2 (9.1)
AMP&FOT&TAZ	1 (4.5)
AMP&FOT&TMP	1 (4.5)

^a^ AMP = ampicillin, AZI = Azithromycin, CEF = Cefotaxim, CIP = ciprofloxacin, CHL = chloramphenicol, GEN = Gentamicin, NAL = Nalidixic Acid, TET=tetracycline, TAZ = Ceftazidime, TMP = trimethoprim, SMX = sulfamethoxazole.

**Table 7:** Overview of the antibiotics screened for in the pig manure.

Antbiotic Residue	Precurson ion (*m*/*z*)		Product ion (*m*/*z*)	RT ^a^ (min)	Antbiotic Residue	Precurson ion (*m*/*z*)		Product ion (*m*/*z*)	RT ^a^ (min)
*β-lactam antibiotics*					*Quinolones*				
amoxicillin	366.01	>	207.93	1	cinoxacin	262.90	>	188.99	4.31
ampicillin	349.95	>	106.02	2.9	nalidixic acid	232.86	>	130.93	4.95
benzylpenicillin	334.94	>	175.95	4.79	oxolinic acid	261.90	>	159.91	4.59
cefalexin	347.93	>	157.97	2.99					
cefalonium	458.98	>	336.91	2.78	*Fluoroquinolones*				
cefapirin	423.90	>	291.94	1.47	ciprofloxacin	331.97	>	230.96	3.55
+metabolite desacetyl cefapirin	381.89	>	111.86	0.82	danofloxacin	357.99	>	82.02	3.71
cefazolin	454.87	>	322.93	3.49	difloxacin	400.04	>	298.97	4.15
cefoperazone	668.40	>	165.00	5.08	enoxacin	321.03	>	205.67	3.34
cefquinome	529.03	>	134.02	2.86	enrofloxacin	360.00	>	245.01	3.8
ceftiofur	523.97	>	240.90	4.95	norfloxacin	319.97	>	232.99	3.46
+metabolite desfuroylceftiofur cystine disulfide	523.97	>	240.90	4.59	flumequine	261.98	>	201.99	5.01
cloxacillin	435.91	>	276.91	5.12	marbofloxacin	362.98	>	72.07	3.29
dicloxacillin	469.99	>	160.11	5.26	sarafloxacin	385.96	>	299.01	4.1
nafcillin	414.97	>	198.97	5.18					
oxacillin	402.01	>	242.93	5.03	*Lincosamides*				
penicillin V	350.97	>	160.02	4.93	lincomycin	407.20	>	126.20	2.4
					pirlimycin	411.07	>	363.05	3.99
*Tetracyclines+epimers*					*Sulfonamides and *				
chlortetracycline	479.02	>	443.93	4.35	*trimethoprim*				
doxycycline	444.99	>	320.98	4.41	sulfachloropyridazine	284.85	>	92.03	3.99
oxytetracycline	460.99	>	425.97	3.32	sulfaclozine	284.92	>	92.03	3.99
tetracycline	444.99	>	409.98	3.6	sulfadiazine	250.89	>	92.03	1.79
					sulfadimethoxine	310.91	>	155.94	4.73
*Macrolides*					sulfadoxine	310.91	>	92.03	4.23
erythromycin A	734.43	>	158.00	4.79	sulfamerazine	264.91	>	107.97	2.65
gamithromycin	777.52	>	619.33	4.58	sulfamethazine	278.92	>	92.03	3.3
spiramycin	843.42	>	174.06	4.38	sulfamethoxazole	253.89	>	92.03	4.25
tilmicosin	869.47	>	174.00	4.65	sulfamethoxypyridazine	280.97	>	92.04	3.49
tulathromycin	806.61	>	577.21	3.92	sulfapyridine	249.90	>	92.04	2.33
tylosin A	916.58	>	174.05	4.85	sulfaquinoxaline	300.91	>	92.03	4.74
tylvalosin	1042.37	>	174.09	5.32	sulfathiazole	255.85	>	108.03	2.25
					trimethoprim	290.98	>	230.01	3.19
*Pleuromutilines*									
tiamulin	494.23	>	192.02	5.03	*Amphenicols*				
valnemulin	565.26	>	263.06	5.09	chloramphenicol	320.85	>	151.85	4.52
					florfenicol	355.78	>	184.87	4.26
*Aminoglycosides*					thiamphenicol	353.85	>	289.89	3.11
apramycin	540.12	>	199.03	6.79	*Diaminopyrimidine*				
	540.12	>	216.98		*derivates*				
	540.12	>	378.00		dapsone	248.90	>	92.03	3.97
dihydrostreptomycin	292.86	>	86.17	5.83					
	292.86	>	176.12		*Polymyxins*				
	292.86	>	263.07		colistin B	578.59	>	101.46	6.54
gentamicin	464.35	>	322.23	6.87		578.59	>	227.92	
(sum of C1, C1a, C2 and C2a)	478.25	>	322.23			578.59	>	570.20	
	450.25	>	322.25						
kanamycin A	485.10	>	163.20	6.57	colistin A	585.56	>	101.46	6.51
	485.10	>	187.20			585.56	>	202.09	
	485.10	>	324.20			585.56	>	576.86	
neomycin B	615.21	>	161.14	6.98	*Internal standards*				
	615.21	>	163.27		cefotaxime	455.90	>	124.99	3.22
	616.42	>	203.23		ceftiofur-d_3_	527.06	>	244.09	4.59
	615.21	>	293.07		chloramphenicol-d_5_	325.85	>	156.85	4.5
	615.21	>	323.19		cincophen	249.92	>	127.98	5.06
paromomycin	616.42	>	163.03	6.79	clindamycin	425.08	>	126.10	4.44
	616.42	>	293.26		lomefloxacine	351.98	>	265.02	3.68
	616.42	>	324.39		methacycline	443.10	>	381.09	4.4
spectinomycin	350.94	>	98.05	3.1	piperacillin	540.00	>	181.94	4.71
	350.94	>	315.00		polymyxin B1	602.54	>	101.03	4.34
	350.94	>	332.91		ribostamycin	455.37	>	295.23	6.51
streptomycin	291.50	>	176.01	5.8	roxithromycin	837.37	>	158.01	5.07
	291.50	>	263.21		sulfadimethoxine ^13^C_6_	316.96	>	98.08	4.73
	291.50	>	295.23		trimethoprim-d_9_	300.04	>	263.98	3.12
tobramycin	468.10	>	162.96	6.9					
	468.10	>	324.05						
	468.10	>	144.95						

^a^: RT: Retention time. For the multi-residue method (all residues except aminoglycosides and polymyxins), a screening method was used, where the transition was followed from the precursor ion to one product ion. In case a signal was obtained, the sample was reinjected and minimum 2 product ions were followed.

**Table 8:** Overview of the limit of detection (LOD), limit of quantification (LOQ), and expanded measurement uncertainty (U) (calculated using 3 concentration ranges, namely 5, 25, and 50 µg/kg, unless specified in table), for the antibiotics tested using the multi-residue method that were detected and quantified (semi-quantitative) in pig manure.

Antibiotic Residue	LOD (µg/kg)	LOQ (µg/kg)	U (%) (k = 2)	Antibiotic Residue	LOD (µg/kg)	LOQ (µg/kg)	U (%) (k = 2)
amoxicillin	35 ^a^	-	-	nalidixic acid	1.3	4.3	20
ampicillin	35 ^a^	-	-	oxolinic acid	1.6	5.5	21
benzylpenicillin	-	-	-	ciprofloxacin	1.6	5.2	31
cloxacillin	75 ^a^	-	-	danofloxacin	1.8	5.8	26
dicloxacillin	-	-	-	difloxacin	2.6	8.5	32
nafcillin	-	-	-	enoxacin	2.1	7.2	40
oxacillin	-	-	-	enrofloxacin	1.9	6.2	44
penicillin V	75 ^a^	-	-	norfloxacin	1.3	4.2	38
cefalexin	6.6	22.2	35	flumequine	0.76	2.5	31
cefalonium	11.6	38.8	60 ^b^	marbofloxacin	1.8	5.8	26
cefapirin	4.5	15.0	22	sarafloxacin	1.3	4.2	29
desacetyl cefapirin	-	-	-	lincomycin	2.7	9.0	33
cefazolin	8.1	27.2	29 ^b^	pirlimycin	2.2	7.3	29
cefoperazone	-	-	-	sulfachloropyridazine	1.8	6.0	12
cefquinome	2.1	7.0	21	sulfaclozine	7.4	24.7	16
ceftiofur	3.4	11.3	38	sulfadiazine	1.1	3.5	21
desfuroyl ceftiofur cysteine disulfide	2.9	9.7	52	sulfadimethoxine	2.0	6.8	16
chlortetracycline	6.0	20	20	sulfadoxine	0.81	2.7	22
doxycycline	5.4	17.9	15	sulfamerazine	1.8	6.0	14
oxytetracycline	3.4	11.3	22	sulfamethazine	0.89	3.0	22
tetracycline	3.0	9.9	43	sulfamethoxazole	1.7	5.8	31
erythromycin	-	-	-	sulfamethoxypyridazine	3.0	10.2	11
gamithromycin	1.8	5.8	34	sulfapyridine	2.3	7.5	16
spiramycin	35^a^	-	-	sulfaquinoxaline	3.2	10.8	16
tilmicosin	2.4	8.0	17	sulfathiazole	3.9	13.0	22
tulathromycin	35^a^	-	-	trimethoprim	1.3	4.3	6
tylosine	5.2	12.7	28	chloramphenicol	5.8	19.3	52
tylvalosin	1.6	5.2	37	florfenicol	3.2	5.3	44
tiamuline	2.5	8.5	20	thiamphenicol	4.9	16.3	69
valnemulin	2.5	8.2	19	dapson	2.5	9.5	20
cinoxacin	1.2	4.2	17				

^a^: LOD was estimated from the chromatograms. ^b^: at 50 µg/kg.

**Table 9:** Overview of the used concentrations during the validation, limit of detection (LOD), limit of quantification (LOQ), intralaboratory reproducibility (RSDR), expanded measurement uncertainty (U), mean linearity (R^2^) for the selected aminoglycosides in pig manure at the different validation levels.

Antibiotic Residue	Spiked Levels for Validation (µg/kg)	LOD (µg/kg)	LOQ (µg/kg)	Repeatability (%)	RSDR (%)	U (%)	Linearity (R^2^)
apramycin	250, 500, 1000	34	113	8	9	29	0.9946
dihydrostreptomycin	250, 500, 1000	10	33	6	8	22	0.9938
gentamicin	250, 500, 1000	21	72	9	8	31	0.9899
kanamycin	250, 500, 1000	73	243	20	26	64	0.9763
neomycin	250, 500, 1000	47	157	11	15	46	0.9885
paromomycin	250, 500, 1000	29	97	6	9	30	0.9954
spectinomycin	1000, 2000, 4000	394	1313	25	55	126	0.9112
streptomycin	500, 1000, 2000	32	107	7	7	22	0.9946
tobramycin	250, 500, 1000	17	57	8	10	35	0.9895
